# An Ensemble Learning Model for Aging Assessment of Silicone Rubber Considering Multifunctional Group Comprehensive Analysis

**DOI:** 10.3390/polym17222988

**Published:** 2025-11-10

**Authors:** Kun Zhang, Chuyan Zhang, Zhenan Zhou, Zheyuan Liu, Yu Deng, Chen Gu, Songsong Zhou, Dongxu Sun, Hongli Liu, Xinzhe Yu

**Affiliations:** 1 School of Artificial Intelligence, China University of Geosciences, Beijing 100190, China; fena1220@email.cugb.edu.cn (K.Z.); zhenanzhou@email.cugb.edu.cn (Z.Z.); liuhongli@email.cugb.edu.cn (H.L.); 2 Tibet Yangbajing High Altitude Electrical Safety and Electromagnetic Environment National Observation and Research Station, Lhasa 851517, China; liuzheyuan@epri.sgcc.com.cn (Z.L.); dengyu@epri.sgcc.com.cn (Y.D.); guchen@epri.sgcc.com.cn (C.G.); zhousongsong@epri.sgcc.com.cn (S.Z.); 3 High Voltage Department, China Electric Power Research Institute, Beijing 100192, China; sundongxu@epri.sgcc.com.cn

**Keywords:** composite insulators, silicone rubber, machine learning, FTIR, aging assessment

## Abstract

With the widespread deployment of high-voltage and ultra-high-voltage transmission lines, composite insulators play a vital role in modern power systems. However, prolonged service leads to material aging, and the current lack of standardized, quantitative methods for evaluating silicone rubber degradation poses significant challenges for condition-based maintenance. To address this measurement gap, we propose a novel aging assessment framework that integrates Fourier Transform Infrared (FTIR) spectroscopy with a measurement-oriented ensemble learning model. FTIR is utilized to extract absorbance peak areas from multiple aging-sensitive functional groups, forming the basis for quantitative evaluation. This work establishes a measurement-driven framework for aging assessment, supported by information-theoretic feature selection to enhance spectral relevance. The dataset is augmented to 4847 samples using linear interpolation to improve generalization. The proposed model employs k-nearest neighbor (KNN), Support Vector Machine (SVM), Random Forest (RF), and Gradient-Boosting Decision Tree (GBDT) within a two-tier ensemble architecture featuring dynamic weight allocation and a class-balanced weighted cross-entropy loss. The model achieves 96.17% accuracy and demonstrates strong robustness under noise and anomaly disturbances. SHAP analysis confirms the resistance to overfitting. This work provides a scalable and reliable method for assessing silicone rubber aging, contributing to the development of intelligent, data-driven diagnostic tools for electrical insulation systems.

## 1. Introduction

With the rapid development of power transmission and distribution systems, especially the construction of high-voltage and ultra-high-voltage transmission lines, composite insulators have gradually become an indispensable key component in power systems. According to statistics from State Grid Corporation, by 2021, the usage of composite insulators in China has exceeded 10 million units [1,2]. However, the silicone rubber skirt material is prone to irreversible aging, such as molecular chain breakage and functional group degradation, when exposed for long periods to coupled stresses such as corona discharge, humidity, heat, and mechanical vibrations. This aging leads to a deterioration in insulating performance or even breakdown accidents, resulting in power system failures, significant economic losses, and safety hazards [3,4,5,6,7]. Therefore, timely and effective assessment of the aging degree of composite insulators is of great significance for the health management, operation maintenance, and preventive testing of power equipment.

Traditional aging detection methods, such as visual inspection and electrical performance testing, generally provide limited aging information and often rely on manual judgment, making it difficult to comprehensively and accurately assess the aging status of composite insulators [8,9,10]. Moreover, most existing aging detection methods are offline, requiring the removal of composite insulators or transporting them to a laboratory for testing, which lacks portability and real-time capabilities, thus hindering on-site monitoring and large-scale evaluation [11,12,13,14].

Fourier Transform Infrared Spectroscopy (FTIR) is a non-destructive and rapid technique that can sensitively capture functional group changes related to silicone rubber aging. Compared with traditional inspection or electrical testing, it provides richer chemical information and more objective assessment, while its quantitative spectral data are highly compatible with machine learning models, enabling accurate and reliable condition evaluation. Some newer studies have applied FTIR technology to the aging research of composite insulators [15,16,17,18,19]. However, most of these studies use FTIR merely as a supplementary analysis tool to verify certain conclusions or examine the changes in a few functional groups, lacking in-depth research on the comprehensive analysis of multiple functional groups. During the aging process, the synergistic changes of multiple functional groups may more accurately reflect the overall aging status of composite insulators. Therefore, how to perform joint analysis of multiple functional groups and incorporate more aging features remains one of the main challenges in current research.

In addition, while machine learning has shown great potential in the aging prediction of composite insulators [20,21,22,23], existing studies often face the issue of insufficient sample sizes. Most studies use small datasets, usually containing only dozens or a few hundred samples, which is insufficient to meet the needs of machine learning model training. This leads to poor generalization ability and low accuracy in the prediction results.

This study presents an automated aging assessment model for composite insulators. To address limited data, a linear interpolation-based augmentation strategy expands the dataset to 4847 samples, enhancing robustness in data-scarce conditions. Key spectral features are extracted using information entropy and mutual information, enabling effective multi-feature coupling analysis. An ensemble learning framework is then employed to optimize classification boundaries and suppress noise. The proposed method achieves 96.17% accuracy in a three-class aging task, significantly outperforming conventional approaches and demonstrating strong potential for accurate aging evaluation.

This model addresses the lack of standardized methods for aging assessment of composite insulators and eliminates the subjectivity of manual evaluation. Providing a solution for large-scale aging assessment of composite insulators.

## 2. Experimental Data

### 2.1. Data Sources

The samples used in this study came from the composite insulators replaced in the operating lines of the provincial companies under the jurisdiction of the State Grid, involving different operating years, different working environments and different aging types. Considering that the most critical property of an insulator is its external insulation performance, a comprehensive evaluation was conducted based on surface condition, hardness (Shore hardness), and hydrophobicity (spray classification method). Based on the evaluation results, the aging degree of silicone rubber was initially classified. It is specifically divided into three aging classes: Light, Medium, and Severe. The detailed classification criteria are shown in Table 1. Among them, new insulators are categorized as slight aging grade.

**Figure 1:**
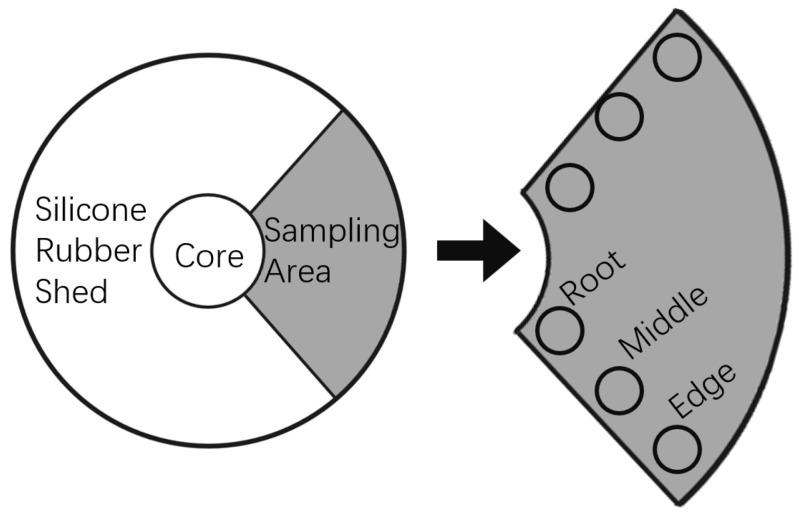
Sampling Schematic.

In the operation of composite insulators, the degree of influence from environmental factors such as sunlight, rainwater, contamination deposition, and electric field distribution varies at different positions of the umbrella skirt. As a result, tests conducted at different locations on the same sample may show significant differences. To improve the representativeness and accuracy of the test results, when testing composite insulators after they have been in service, 4 to 6 different locations are selected from each umbrella skirt for testing based on the skirt’s size. The sampling locations are chosen to cover the root, middle, and edge of the umbrella skirt as much as possible, as shown in Figure 1. For new composite insulator samples, since they have not yet been in operation and have not undergone uneven aging on the surface, only one random point on the surface is selected for testing.

Hardness testing was conducted using a Shore A durometer, which directly measures the surface hardness of the silicone rubber samples, providing an indication of their material rigidity. FTIR spectroscopy was performed using a Thermo Scientific Nicolet iS50 (Manufacturer: Thermo Fisher Scientific, Waltham, MA, USA), with ATR (Attenuated Total Reflectance) as the testing method. The equipment was equipped with a diamond crystal and a DTGS KBr detector (Manufacturer: Thermo Fisher Scientific, Waltham, MA, USA). The spectra were collected with 32 scans, within a wave number range of 550–4000 cm^−1^ and a resolution of 4 cm^−1^. To ensure the accuracy of the acquired spectral data, a fresh background spectrum was collected every 15 min during the FTIR testing. After acquisition, the raw spectra were automatic subjected to baseline correction using OMNIC software (Version:9.7.46) to minimize potential baseline drift. Considering the stable laboratory environment, the high performance of the instrument, and the smooth shapes of the obtained spectra without evident spikes or noise, no additional smoothing procedures were applied. A total of 740 samples were collected.

### 2.2. Selection of Characteristic Functional Groups

To conduct quantitative analysis of the FTIR spectra, it is essential to first identify the characteristic functional groups related to the aging process of silicone rubber. This study identifies the following functional groups as being related to the aging of silicone rubber: silimethyl (Si-CH_3_), siloxane backbone (Si-O-Si), hydrocarbon bonds stretching vibration (C-H_SV), hydrocarbon bonds bending vibration (C-H_BV) and hydroxyl group (-OH) [24,25,26,27,28].

The absorption peak wave numbers of each group are shown in Table 2:

### 2.3. Selection of Aging Characteristics

To quantify the discriminative ability and predictive relevance of each feature, this study employs information entropy and mutual information [29] to evaluate the relationship between features and target variables. This allows for the identification of the most contributive features to the prediction task, while eliminating redundant and irrelevant features to improve model efficiency and accuracy.

Information entropy is used to measure uncertainty and reflect the degree of dispersion of a particular feature. In this study, information entropy is applied to assess the diversity of each feature and the amount of information it provides. The definition of information entropy is based on information theory and was proposed by Shannon. The formula is as follows:

(1) $$H\left(X\right)=-\sum_{i=1}^{n}p\left(x_{i}\right)\log_{2}p\left(x_{i}\right)$$

where H(X) represents the entropy of the random variable *X*, $x_{i}$ represents the possible values that the feature *X* can take. $p(x_{i})$ is the probability distribution of $x_{i}$. A higher entropy value indicates that the feature has greater information content, meaning the feature is more capable of distinguishing different samples.

Through mutual information, the relationship between two variables and their mutual dependence can be assessed. Mutual information reveals how much a feature contains information about the target variable. Alternatively, it can also be interpreted as the amount of information the target variable holds about the feature. The higher the mutual information, the stronger the correlation between the feature and the target variable, meaning the feature contributes more to predicting the target variable. The formula is as follows:

(2) $$I\left(X,Y\right)=\sum_{i=1}^{n}\sum_{j=1}^{m}p\left(x_{i},y_{j}\right)\log_{2}\frac{p\left(x_{i},y_{j}\right)}{p\left(x_{i}\right)p\left(y_{j}\right)}$$

where I(X,Y) is the mutual information between feature *X* and target variable *Y*. $p(x_{i},y_{i})$ is the probability of co-occurrence of feature *X* and target variable *Y*. $p(x_{i})$ and $p(y_{i})$ are the marginal probability distributions of *X* and *Y*.

**Figure 2:**
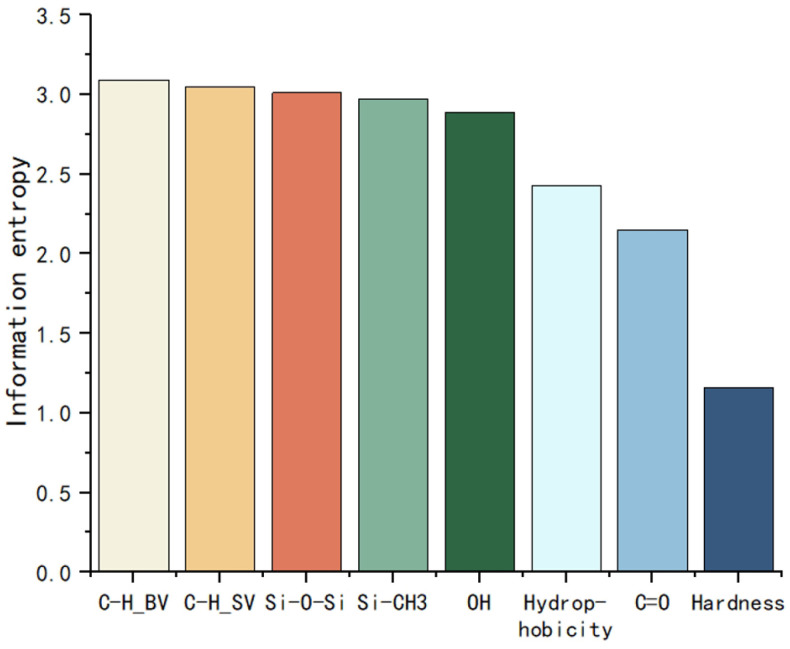
Information entropy of each feature.

**Figure 3:**
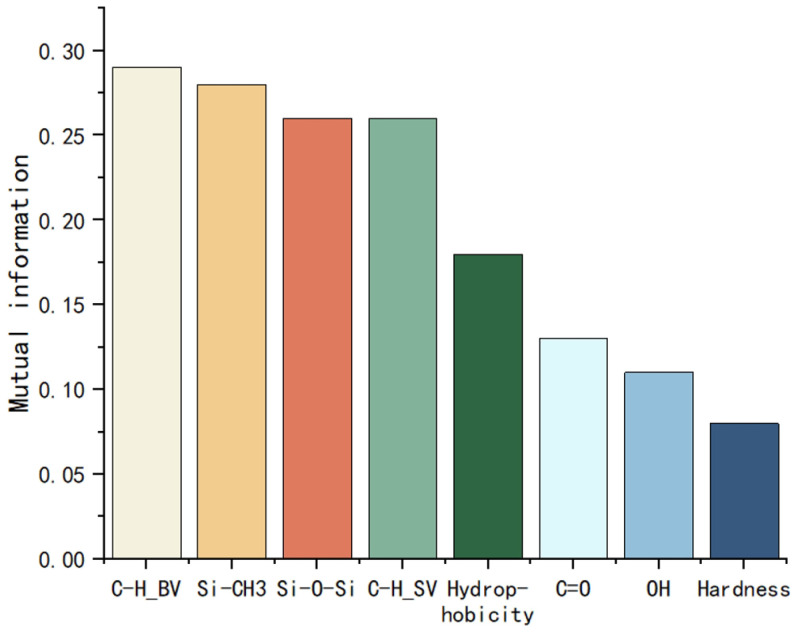
Mutual information of each feature.

By calculating the mutual information, the correlation between each feature and the degree of aging can be initially assessed quantitatively. The information entropy and mutual information of each feature are shown in Figure 2 and Figure 3.

Based on the results, this study selects Si-CH_3_, Si-O-Si, C-H bending vibration, C-H stretching vibration, and hydroxyl group as the input features.

### 2.4. Data Augmentation

During the construction of the silicone rubber aging state evaluation model, although the original dataset contains 740 samples, the demand for data volume in a complex three-classification model makes the current sample size insufficient for model training. This may lead to underfitting and a decrease in classification performance.

To address this issue, this study uses linear interpolation for data augmentation, generating new samples based on the existing data, thus expanding the dataset size. FTIR spectra reflect the chemical molecular vibration modes, typically represented by absorption peaks at different wave numbers. Linear interpolation generates new data points through a simple linear transition between adjacent points. Considering that the model is trained using the area of functional group absorption peaks, and the impact of spectral nonlinearity caused by linear interpolation on the area is minimal, it is believed that data augmentation can be performed using this method.

Assuming that two samples, $x_{i}=\left(x_{i1},x_{i2},...,x_{in}\right)$ and $x_{j}=\left(x_{j1},x_{j2},...,x_{jn}\right)$ from the original dataset have corresponding labels $y_{i}$ and $y_{j}$. New samples can be generated by linear interpolation and can be represented as:

(3) $$x_{new}=\alpha x_{i}+\left(1-\alpha \right)x_{j},\;\alpha \in \left[0,1\right]$$

(4) $$y_{new}=\alpha y_{i}+\left(1-\alpha \right)y_{j},\;\alpha \in \left[0,1\right]$$

During data augmentation, to prevent the combination of samples with significant differences from producing anomalous results, augmented samples are all taken from different locations on the same insulator sleeve. These samples experience the same types of environmental effects during aging, with the only difference being the intensity. As a result, the aging mechanisms are the same, leading to similar spectral shapes with the main differences occurring in peak intensity. Therefore, the newly generated samples will inevitably be at the same aging level as the original samples, without any obvious anomalies.

Where α is the weight factor. By adjusting the value of α, a series of new samples can be generated, increasing the sample size and enhancing the model’s generalization ability for spectral data while avoiding overfitting. After augmentation, a total of 4847 data samples are obtained, with 1036 lightly aged samples, 1552 moderately aged samples, and 2259 severely aged samples.

## 3. Model Architecture Design

### 3.1. Model Introduction

Classification problems are one of the core tasks in machine learning. The objective is to learn a function f:X→Y when given a set of input data *X* and corresponding category labels *Y* (discrete variables), enabling the model to accurately predict the category $Y^{\prime }$ of unknown data $X^{\prime }$. Specifically, assume that the input data is $X=\left\{x_{1},x_{2},...,x_{n}\right\}$, the corresponding label is $Y=\left\{y_{1},y_{2},...,y_{n}\right\}$ and the model’s prediction result is $\hat{y}$. The goal of machine learning is to find the optimal parameters $\theta^{*}$, minimizing the loss function $L\left(y,\hat{y}\right)$. Mathematically, it is expressed as:

(5) $$\theta^{*}=arg\min_{\theta }\frac{1}{n}\sum_{i=1}^{n}L\left(f\left(x_{i};\theta \right),y_{i}\right)$$

In the process of constructing an aging assessment model for silicone rubber, considering the possibility of future integration into portable handheld devices and the relatively small sample size, we opted for machine learning models, which are more suitable for small datasets and can deliver both efficiency and practical applicability in real-world scenarios. However, while the use of a single machine learning model offers high computational efficiency, it often fails to perform a comprehensive analysis of multiple features due to structural limitations, resulting in an inability to capture the subtle characteristics of the aging process.

To address the respective limitations of both simple and complex models, this study introduces the concept of ensemble learning. Ensemble learning enhances prediction performance by integrating the outputs of multiple base learners, thereby leveraging the strengths of each individual model. Simpler models gain improved expressive power through ensemble integration, while more complex models benefit from reduced overfitting within the ensemble framework. By combining predictions from multiple learners, ensemble methods not only maintain high predictive accuracy but also significantly improve model stability and generalization capability, enabling more precise assessment of silicone rubber aging.

**Figure 4:**
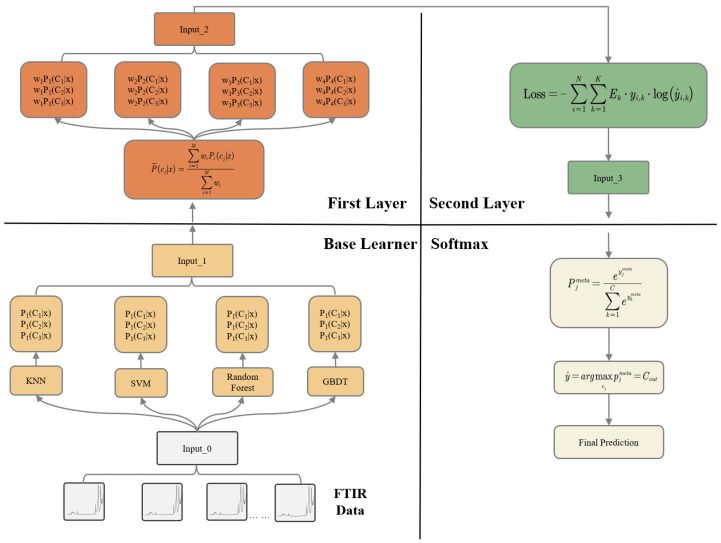
Model structure.

In the proposed approach, the FTIR peak-area features are first fed into four heterogeneous base learners (KNN, SVM, RF, and GBDT), each of which independently outputs class probabilities for the three aging levels. These probability outputs are then passed to a first fusion layer that applies a weighted voting mechanism to assign an adaptive weight to each base learner according to its contribution. The weighted probability vectors are further provided to a second fusion layer based on a stacking strategy, where a meta-learner integrates the complementary information from different models under a class-balanced loss. Finally, the fused logits are converted into the final aging class through a Softmax function. The overall model architecture is shown in Figure 4.

### 3.2. Base Learners

The model first utilizes four base learners for prediction, processing the results to obtain their predicted probabilities. Among these, KNN (k-Nearest Neighbors) is an instance-based learning algorithm suitable for handling data with local structures. Since FTIR spectral data may exhibit local patterns or regularities, KNN is capable of making reasonable classification predictions by identifying historical data points similar to the test data.

SVM (Support Vector Machine) maps data to a high-dimensional space using a kernel function to achieve better classification boundaries. Therefore, when dealing with complex FTIR spectral data, SVM can effectively identify the decision boundaries of the aging states.

Random Forest (RF), a classic method in ensemble learning, is known for its strong overfitting resistance. It has a built-in feature selection mechanism, which helps eliminate redundant or irrelevant features.

Gradient-Boosting Decision Tree (GBDT) is an iterative additive model that enhances model performance by continuously optimizing residuals. When handling complex nonlinear relationships, GBDT can progressively improve prediction results. In the context of composite insulator aging prediction, it is particularly sensitive to subtle changes.

For each base learner, we performed automated hyperparameter tuning with Optuna using the TPE sampler with pruning. Trials were assessed by stratified K-fold crossvalidation with macro-F1 as the objective; the best configuration was then refit on the full training set before final testing. The selected hyperparameters for all models are summarized in Table 3.

### 3.3. First Layer

After the base learners have completed their predictions, the predicted probabilities are used as inputs for the first layer of the model. In this layer, the Voting algorithm is employed to weight the predicted probabilities from each base learner, thereby determining the weight of each model. The combined prediction probability for each class is calculated as follows:

(6) $$\bar{P}\left(c_{j}|x\right)=\frac{\sum_{i=1}^{M}\omega_{i}P_{i}\left(c_{j}|x\right)}{\sum_{i=1}^{M}\omega_{i}}$$

Among them, $\bar{P}\left(c_{j}|x\right)$ is the weighted comprehensive prediction probability of category $c_{j}$, $\omega_{i}$ is the weight of each base learner, and the denominator $\sum_{i=1}^{M}\omega_{i}$ serves to ensure the normalization of the weights.

Unlike traditional Voting methods, this layer does not directly output the final fused result. Instead, it assigns the optimal weights to the predicted probabilities of the corresponding base learners. These weighted predicted probabilities are then used as inputs for the second layer of the model, where further fusion takes place. This approach allows the model to fully account for the contribution of each base learner while ensuring that, during the second-layer fusion, the outputs of the base learners are effectively combined. As a result, this method provides a more accurate basis for the final prediction.

### 3.4. Second Layer

The second layer of the model primarily utilizes the Stacking algorithm to fuse the weighted predicted probabilities from the first layer. Stacking enhances predictive performance by using the outputs of multiple base learners as new input features, which are then passed to a meta-learner for secondary learning. In this study, the first layer assigns optimal weights to the predicted probabilities of each base learner using the Voting algorithm. These weighted predictions are then provided as inputs to the second layer, offering the meta-learner more refined feature information. This approach enables the second-layer model to optimize the predictions of each base learner at a higher class, improving the overall fusion effect.

In classification tasks, meta-learners typically use a cross-entropy loss function. However, in aging classification tasks, the distribution of samples across different aging class may be uneven, which can lead to poor predictive performance for certain categories when using the traditional cross-entropy loss function.

To address this issue, this study proposes modifying the meta-learner’s loss function to a weighted cross-entropy loss function during the implementation of Stacking. The weighted cross-entropy loss function, compared to the standard cross-entropy loss, adjusts the weights of different classes based on the importance or distribution balance of each class, thereby optimizing the model’s training process.

First, the weighted average error rate for each base learner in each class is computed:

(7) $$E_{k}=\sum_{m=1}^{M}\omega_{m}\cdot \frac{e_{m,k}}{t_{k}}$$

where $e_{m,k}$ denotes the number of prediction errors of the m-th base learner on category *k*, $t_{k}$ denotes the total number of samples in category *k*, and $\omega_{m}$ denotes the weight of the *m*-th base learner.

After obtaining the weighted average error rate $E_{K}$, it is incorporated into the crossentropy loss. The loss function is modified as follows:

(8) $$\mathrm{Loss}=-\sum_{i=1}^{N}\sum_{k=1}^{K}E_{k}\cdot y_{i,k}\cdot \log\left({\hat{y}}_{i,k}\right)$$

where *N* is the number of samples, *K* is the number of classes, $y_{i},k$ is the true label of sample *i* for class *k* (one-hot encoding, with the true class being 1 and all others 0), and ${\hat{y}}_{i,k}$ is the predicted probability of sample *i* for class *k*.

By incorporating the weighted cross-entropy loss function, the meta-learner is able to focus more on the samples from difficult-to-predict classes during training, thereby improving classification performance and enhancing the overall prediction effectiveness.

### 3.5. Model Output

In this study, the Softmax function is used in the final output layer of the model to convert the prediction results into a probability distribution. The Softmax function normalizes the scores for each class in the model’s output and transforms them into a non-negative probability distribution, as shown in Equation (9).

(9) $$soft\max\left(z_{i}\right)=\frac{e^{z_{i}}}{\sum_{j=1}^{n}e^{z_{j}}}$$

where $z_{i}$ is the i-th element in the input vector, $e^{z_{i}}$ is the natural exponential function, and the denominator part is the exponential sum of all input values.

The use of Softmax offers two significant advantages. First, it converts the raw scores from the model into interpretable probability values, allowing each class’s prediction to be clearly represented as a probability. This is crucial for decision support systems, as it enables more informed decision-making based on the probability values for each class.

Second, the Softmax function enhances the model’s ability to distinguish between multiple classes by normalizing the predicted scores for each class. In multi-class classification tasks, especially when the differences between classes are small or there is significant overlap, Softmax effectively focuses the model’s attention on the most probable class, thereby improving prediction accuracy. Furthermore, the probability outputs from Softmax allow the model to better reflect its understanding of the relative importance of different classes, particularly in cases of class imbalance or ambiguous boundaries. This further enhances the model’s robustness and generalization capability.

## 4. Training and Testing

### 4.1. Model Training

The absorption peak areas of five functional groups for all samples after data augmentation are calculated and used as inputs for the model. The samples are then split into a training set and a testing set in a 0.8:0.2 ratio, and the base learners are trained.

During the training of the base learners, K-fold cross-validation is used to improve the model’s generalization ability, fully utilize the dataset, and further address the issue of class imbalance. Specifically, the dataset is divided into *K* equally sized subsets, with each subset referred to as a “fold”. In K-fold cross-validation, the training and validation process is repeated *K* times, with each fold serving as the validation set once, while the remaining K−1 folds are used to train the model.

**Figure 5:**
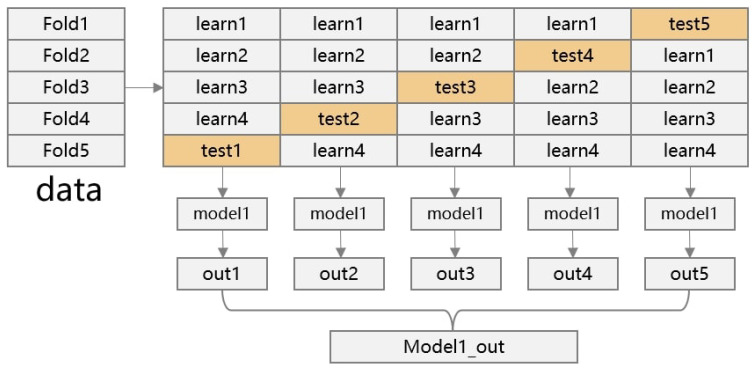
K-fold cross-validation.

Additionally, by selecting different types of algorithms, the approach ensures that their prediction results are complementary, reducing the bias and variance associated with a single model. The cross-validation process for the base learners is illustrated in Figure 5.

### 4.2. Model Performance

Upon completion of the base learner training, the individual base learners are fused to form a new ensemble learning model. The model’s accuracy on the test set, along with precision, recall, and F1 score for each aging class, are computed, as presented in Table 4. The calculation methods for these performance metrics are outlined as follows:

(10) $$\begin{array}{c} \begin{array}{cc} A & =\frac{T}{T+F}\\ P_{i} & =\frac{T}{T+F_{P}}\\ R_{i} & =\frac{T}{T+F_{N}}\\ F_{1i} & =\frac{2P_{i}PR_{i}R}{P_{i}P+R_{i}R} \end{array} \end{array}$$

where *T* represents the number of correctly predicted samples, *F* is the number of incorrectly predicted samples, $F_{p}$ is the number of negative categories that were incorrectly predicted as positive, and $F_{N}$ is the number of positive categories that were incorrectly predicted as negative.

**Figure 6:**
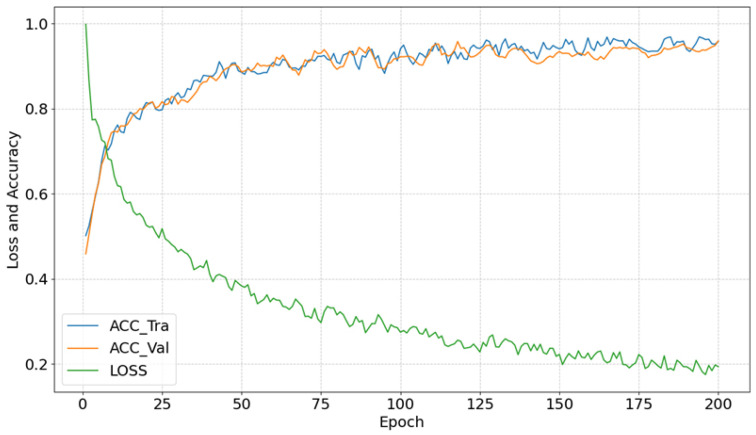
Model Loss and Accuracy Curves.

Furthermore, to assess the model’s performance, the loss and accuracy curves during the training process are plotted, as shown in Figure 6.

An analysis of the metrics in Table 4 reveals that the overall performance of the model is outstanding. Both the precision and F1 scores are high, indicating that the model is able to classify each category with considerable accuracy, resulting in a low proportion of misclassifications. Recall rates show slight variation, with the first category exhibiting a lower recall. This discrepancy may be attributed to the limited number of samples in this category or the less distinctive features associated with it, which could diminish the model’s recognition capability for this class. Overall, the accuracy is remarkably high, reaching 96.17%, further validating the model’s superior performance in the task at hand.

As illustrated in Figure 6, during the early stages of training, both the loss and accuracy gradually improve as the training progresses. This suggests that, in the initial phase, the model is effectively minimizing prediction errors and classifying data correctly by finetuning its parameters. In the later stages of training, both accuracy and loss levels stabilize, with accuracy reaching approximately 96% and loss decreasing below 20%, indicating that the model has achieved a satisfactory level of learning and has converged.

**Figure 7:**
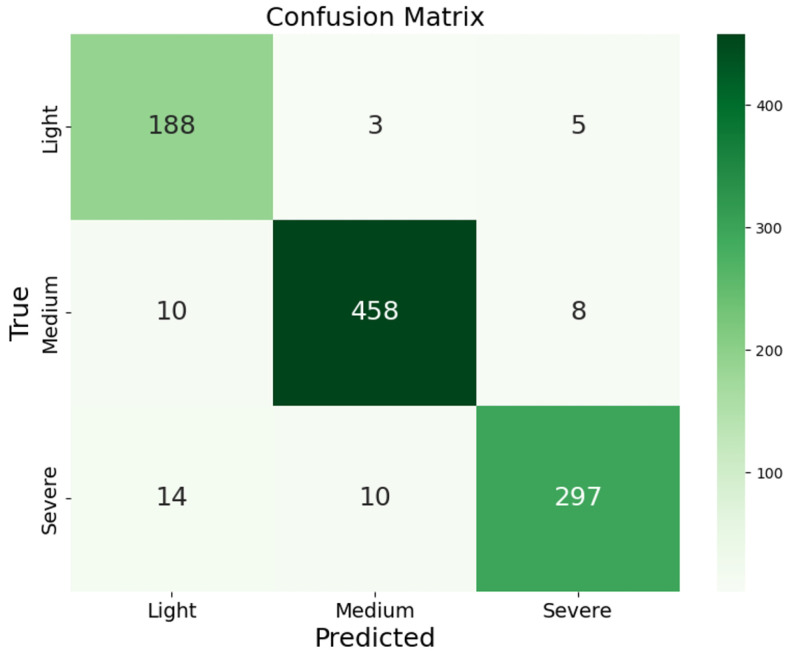
Confusion Matrix.

To evaluate the model’s classification capabilities and to assess its performance on each class, a confusion matrix is employed to display the model’s predictions across different class labels. This enables a thorough analysis of the model’s strengths and areas that require improvement. The confusion matrix for the ensemble learning model trained in this study is presented in Figure 7.

It can be observed that the model performs optimally in the moderate aging category, correctly predicting the majority of the samples with minimal misclassifications. However, for the light and severe aging categories, although the model is able to correctly predict most samples, the misclassification rate is slightly higher compared to the moderate aging category. These misclassifications indicate that the model encounters difficulties in distinguishing between light and severe aging samples. Overall, the model exhibits strong performance, effectively identifying various aging classes; however, there remains room for improvement in reducing misclassifications between light and severe aging categories.

## 5. Model Evaluation

### 5.1. Generalization Capability Evaluation

To assess generalization and diagnose potential overfitting in a multi-class setting, we combine SHAP (Shapley Additive Explanations) with the point-biserial correlation coefficient (PBCC). SHAP provides per-sample, per-feature attributions that quantify how each feature contributes to the model’s predicted class probability, thereby enabling instance-level interpretability. Our diagnostic links these continuous attributions to a label-agnostic correctness indicator $z_{i}\in 0,1$ (1 if sample *i* is correctly classified, 0 otherwise). Intuitively, if the model has learned feature-decision relationships that truly generalize, features with larger supportive SHAP values in training should also be associated with correct decisions in testing; when overfitting occurs, this association weakens or becomes unstable on unseen data [30,31].

Formally, for each feature *j* we compute PBCC between its SHAP values $x_{ij}$ and correctness $z_{i}$. PBCC is Pearson’s correlation specialized to one continuous and one binary variable and equals the standardized mean difference between the “correct” and “incorrect” groups:

(11) $$r_{pb}\left(j\right)=\frac{\mu_{1,j}-\mu_{0,j}}{s_{j}}\sqrt{pq}\;\mathrm{with}\;p=\mathrm{Pr}\left(z=1\right),q=1-p$$

where $\mu_{1}$ and $\mu_{0}$ are the means of $x_{ij}$ in the correct and incorrect groups, and $s_{j}$ is the pooled standard deviation of $x_{ij}$. This construction is well-suited to multi-class problems because “correctness” is defined for every sample independent of its class, yielding a single, comparable measure across classes and avoiding sign ambiguities in multi-class SHAP space.

**Figure 8:**
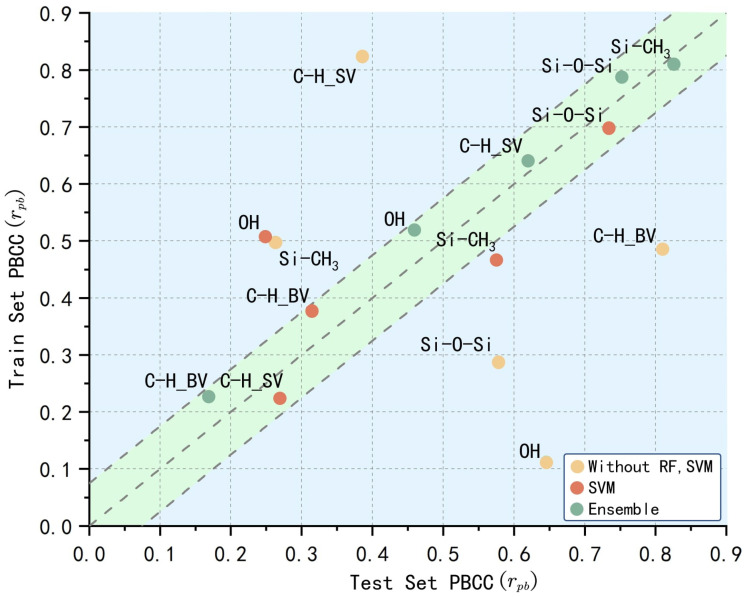
Over- and under-fitting test for different models.

In practice, we compute $r_{pb}$ in the training and testing sets and examine their train–test agreement. Stable agreement indicates that feature attributions supporting decisions are not merely memorizing training idiosyncrasies, whereas large discrepancies signal potential overfitting. The result is shown in Figure 8.

In the figure, the green points represent the ensemble model excluding SVM and RF, the blue points represent the optimized SVM model among the base learners, and the orange points represent the model proposed in this study. The green region indicates the absence of overfitting, while the blue region suggests the potential occurrence of overfitting. It can be observed that most of the features in the proposed model are distributed near the main diagonal within the green region, indicating that the model performs consistently across both the training and testing sets. In contrast, the points for the model excluding SVM and RF deviate significantly from the main diagonal, with the SVM model’s points, particularly those for the hydroxyl group, deviating more drastically. This suggests that the model may have difficulty correctly learning the aging pattern associated with a single functional group, such as the hydroxyl feature, which aligns with the fact that the aging of silicone rubber cannot be easily discerned from the changes in any single functional group alone. The complex and multifaceted nature of silicone rubber aging means that relying on a single feature’s variation is often insufficient to capture the full scope of degradation.

To quantify this metric, this study utilizes the method of least squares to minimize the sum of squared errors and find the optimal fitting line. The performance of each data group is then assessed based on the residuals from the fitting. The smaller the residuals, the closer the data points are to the main diagonal, and the better the model’s performance.

The goal of the fitting error in the least squares method is to minimize the following objective function:

(12) $$E\left(m,b\right)=\sum_{i=1}^{n}{\left(y_{i}-\left(mx_{i}+b\right)\right)}^{2}$$

where $x_{i}$ and $y_{i}$ are are the coordinates of each set of data points and $mx_{i}+b$ is the predicted value of the point on the fitted line.

After obtaining the fitted line, the sum of squares for error (SSE) of the residuals from each point to the fitted line was calculated as shown in Table 5.

From the data in the table, it is evident that the ensemble model has the best fitting performance, with its performance significantly surpassing that of other models, indicating that overfitting or underfitting is unlikely to have occurred. On the other hand, the model excluding SVM and RF shows a higher SSE, with points deviating substantially from the main diagonal, suggesting that it has likely suffered from severe overfitting or underfitting.

Additionally, 90 extra samples were collected for testing, which were not involved in the model training process. After testing, it was found that the model’s prediction accuracy for these samples also reached around 95%, indicating that the model has a strong generalization ability.

### 5.2. Robustness Testing

In practical applications, machine learning models are often required to handle anomalous data, noise, and other perturbing factors. Therefore, evaluating the robustness of a model—specifically its stability and adaptability under various perturbation conditions—is a crucial step in verifying its performance in real-world applications. This study tests the proposed model’s performance under different types of disturbances by introducing perturbations into the input data, thereby assessing its reliability in an imperfect data environment.

This research employs two types of disturbances: anomalous data and Gaussian noise. During the aging process of composite insulators, certain functional groups may undergo abnormal changes due to extreme environmental conditions. The content of these groups may deviate significantly from the normal range due to external factors. To simulate these extreme conditions, we artificially introduce outliers into the input data to examine the model’s stability and adaptability when confronted with anomalous data. The method for introducing anomalous data is as follows:

(13) $$A_{i}^{\prime }=A_{i}\cdot k$$

**Figure 9:**
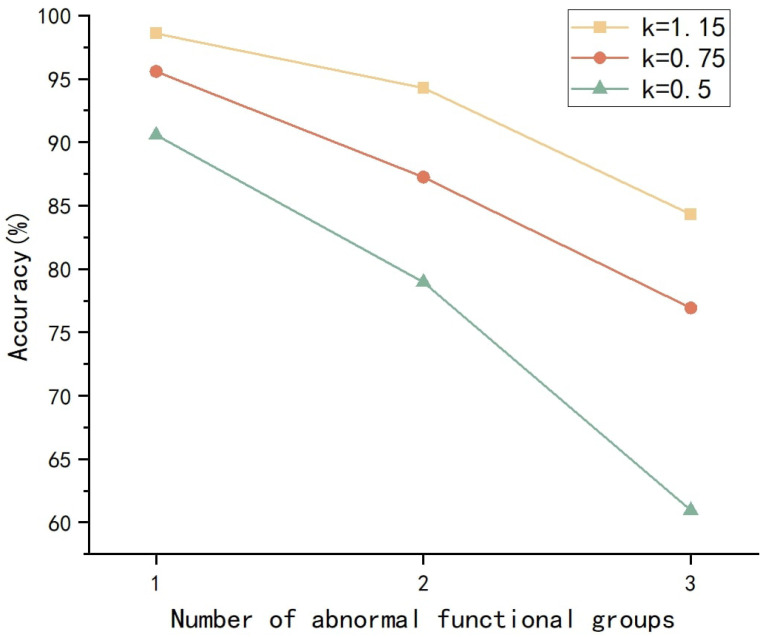
Model performance in the presence of anomalous functional groups.

Here, $A_{i}$ represents the original area of the functional group, and *k* denotes the anomaly coefficient. Considering that significant changes in the area of a functional group are rare in practical environments, the anomaly coefficients are set to 1.15, 0.75, and 0.5. A total of 300 correctly classified samples are selected to test the model’s accuracy when one to three functional groups exhibit anomalies. The results are shown in Figure 9.

It can be observed that, when a single functional group exhibits anomalies, the model maintains a good level of predictive capability, with accuracy remaining above 90% for all three different *k* values. When two functional groups are anomalous, the model’s accuracy significantly decreases, yet it still manages to maintain approximately 80% predictive capability. When three functional groups are anomalous, and *k* = 0.5, the model’s predictive performance is severely impaired, rendering it unable to complete the prediction task. Nevertheless, overall, the model demonstrates good resistance to anomalous data and is able to withstand most abnormal conditions encountered in natural settings.

In FTIR testing of composite insulators, variations in the external environment and the inherent errors of the testing instruments can introduce noise into the spectral signals. This noise typically manifests as random fluctuations correlated with the signal and may affect the accuracy and interpretability of the spectral data. To simulate this phenomenon, this study introduces Gaussian noise into the input data to examine the model’s stability and predictive performance under noisy conditions.

**Figure 10:**
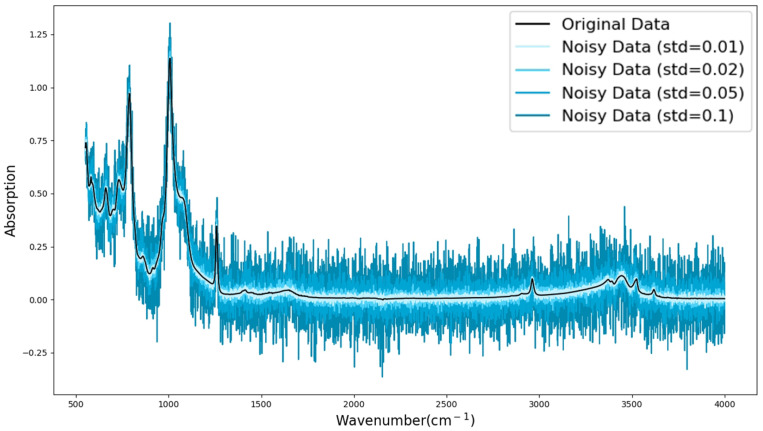
Data with noise added.

Gaussian noise with a mean of zero and a standard deviation set to a specified value is added to the feature values of the FTIR spectrum to simulate signal deviations caused by instrument errors, environmental fluctuations, and other factors. Specifically, the noise is introduced into the spectral data as follows:

(14) $$x_{noisy}=x+\epsilon ,\;\epsilon \;\left(0,\sigma^{2}\right)$$

where *x* is the original FTIR spectrum, ϵ is the Gaussian noise, which follows a normal distribution, and σ is the standard deviation of the noise. The σ is set to four levels of 0.01, 0.02, 0.05 and 0.1, and the FTIR spectra of different noise levels are shown in Figure 10.

**Figure 11:**
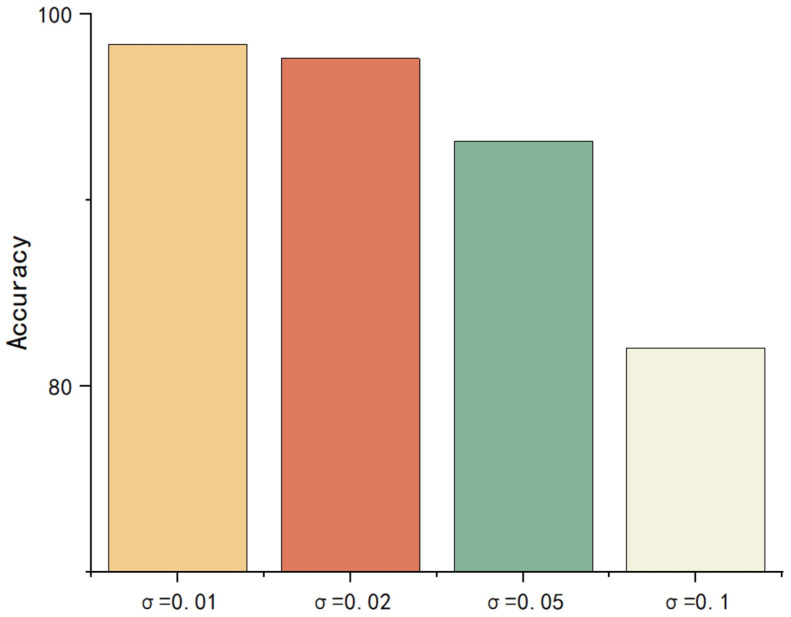
Model accuracy with different noise.

After adding noise to the FTIR spectra, the absorption peak area was recalculated and then the ensemble model was used to predict the aging class. The results are shown in Figure 11:

It can be observed that the model maintains excellent predictive performance when the noise standard deviation, σ, does not exceed 0.05. When σ increases to 0.1, the model’s accuracy experiences a significant decline, but it still remains above 80%. This suggests that despite the increased noise intensity, the model retains strong robustness and is capable of effectively resisting a certain level of noise interference. This result further validates the model’s stability in the face of disturbances of varying intensities, demonstrating its ability to adapt to fluctuations in data quality in practical applications.

### 5.3. Ablation Experiments

To evaluate component contributions within the ensemble model, a series of ablation experiments were conducted. By removing base learners, data augmentation, and weight analysis in turn, the study assessed their individual impact on performance. This analysis identified key elements driving predictive accuracy and model robustness.

For each round of the ablation experiments, one or two base learners were removed, and ensemble models excluding these base learners were constructed. By comparing the changes in model performance before and after the removal of base learners, the contribution of each base learner to the overall model performance was evaluated.

Considering the impact of the model’s initial fusion stage on the ensemble learning effectiveness, the effect of a single fusion process was also separately tested. Specifically, the steps involving the removal of the voting analysis of model weights and the loss function optimization process were excluded to observe the influence of these steps on the final prediction performance.

The results are shown in Table 6.

Ablation experiments confirm the key components that drive the ensemble’s performance. Data augmentation is critical: removing it reduces accuracy to 83.10% (–13.07%), indicating the original sample pool is insufficient for reliable training. Among base learners, GBDT contributes most (its removal causes a 3.53% drop), followed by RF (–2.88%); both excel at modelling nonlinear feature interactions. KNN and SVM have smaller individual effects but improve ensemble diversity by capturing local patterns and boundary information. Removing any two base learners produces a substantial accuracy loss, underscoring the benefit of heterogeneous learners for bias–variance reduction. Finally, the first-layer weight analysis and the optimized loss function are also important: their removal reduces accuracy by 8.42% and 10.29%, respectively, showing that adaptive weighting and loss design materially enhance fusion effectiveness. Overall, GBDT/RF drive most gains, while KNN/SVM, weight analysis, and loss optimization jointly ensure robustness and generalizability.

### 5.4. Performance Comparison

#### 5.4.1. Comparison of Different Machine Learning Models

To comprehensively evaluate the performance and advantages of the proposed ensemble learning model, this study further compares the ensemble model with several classical machine learning models. Through comparative experiments, the relative superiority of the ensemble model in the aging prediction task of composite insulators is validated, and the effectiveness and reliability of ensemble learning methods in practical applications are explored.

**Figure 12:**
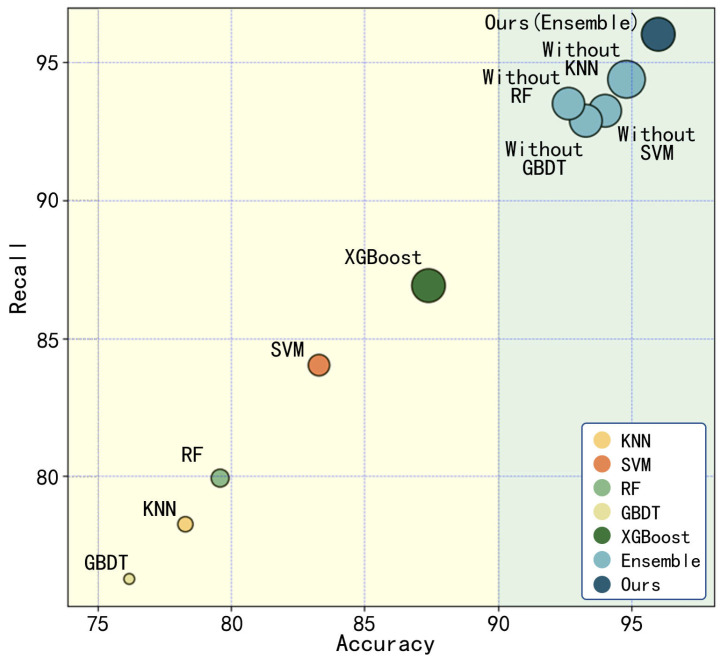
Model comparison results.

The accuracy, recall, and F1-score of the four base learners, XGBoost, the ensemble learning model, and the ensemble learning model with each of the four base learners removed were statistically analyzed, as shown in Figure 12.

In the figure, the horizontal axis represents accuracy, the vertical axis represents recall, the size of the points corresponds to the F1-score, and different colored points represent different models.

The results demonstrate that the performance of the four base learners and XGBoost alone in all evaluation metrics is inferior to that of the proposed ensemble learning model. This finding indicates that the ensemble learning framework can effectively leverage the strengths of different base learners, enhancing overall model performance by integrating predictions from multiple learners. Compared to single models, ensemble learning significantly improves predictive performance.

Furthermore, even when one base learner is removed from the ensemble model, the remaining ensemble still outperforms other single models. Notably, the performance degradation of the ensemble model after removing any single base learner is relatively minor, suggesting that the constructed ensemble framework exhibits strong fault tolerance and robustness.

#### 5.4.2. Comparison of Different Loss Functions

To further investigate the impact of the proposed weighted cross-entropy loss function on the performance of the composite insulator aging prediction model, this study compares two other commonly used loss functions—cross-entropy loss and focal loss—with the weighted cross-entropy loss on datasets of varying scales. Through comparative experiments, the effectiveness of different loss functions in handling the composite insulator aging prediction task can be evaluated. Additionally, this approach helps assess the performance of different loss functions under varying data volumes, thereby validating their robustness, generalization ability, and training stability. The mathematical formulations of cross-entropy loss and focal loss are presented in Equations (15) and (16), respectively.

(15) $$L_{CE}=-\sum_{i=1}^{C}y_{i}\log\left(p_{i}\right)$$

where $y_{i}$ represents the one-hot encoded true label, and $p_{i}$ denotes the model’s predicted probability for class *i*.

(16) $$L_{FL}=-\sum_{i=1}^{C}\alpha_{i}{\left(1-p_{i}\right)}^{\gamma }\log\left(p_{i}\right)$$

where $p_{i}$ is the model’s predicted probability for class *i*, $y_{i}$ is the one-hot encoded true label, $\alpha_{i}$ is the weighting factor for class *i*. γ is a modulating factor used to adjust the influence of easily classified samples.

**Figure 13:**
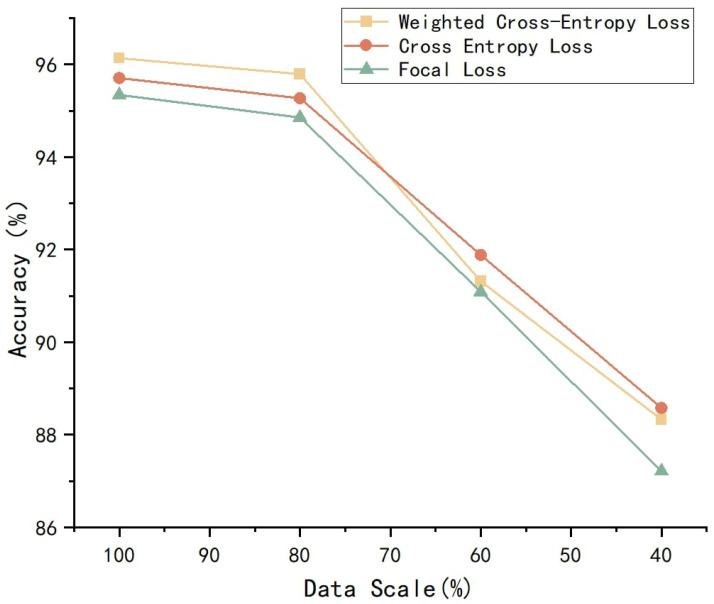
Accuracy of different loss functions.

Three different loss functions are used to train on datasets of different sizes of 100%, 80%, 60% and 40%. The results are shown in Figure 13:

The accuracy of the three loss functions exhibits a consistent pattern across different dataset sizes. As the dataset size decreases, the accuracy of all three loss functions generally declines. Specifically, when the dataset is small, the focal loss demonstrates superior performance, achieving higher accuracy than the other two loss functions. This suggests that focal loss is more effective in handling class imbalance by enhancing the model’s ability to recognize hard-to-classify samples.

Conversely, as the dataset size increases, the weighted cross-entropy loss gradually outperforms both focal loss and standard cross-entropy loss. This indicates that weighted cross-entropy loss can better balance class weights in large-scale datasets, thereby improving overall predictive performance.

Given the relatively large dataset size in this study, the weighted cross-entropy loss is the most appropriate choice. By assigning different weights to different classes, it effectively mitigates the impact of class imbalance while demonstrating strong robustness and generalization ability under large-scale data conditions. Therefore, it serves as the optimal loss function for this research.

## 6. Conclusions

In this study, we propose a measurement-driven aging assessment framework for composite insulators integrating FTIR spectroscopy with an ensemble learning model. By identifying five aging-relevant functional groups through information entropy and mutual information, we construct a two-tier ensemble model. The framework enhances sensitivity and robustness by combining dynamic weight allocation and weighted crossentropy optimization, achieving 96.17% accuracy in ternary classification and maintaining over 95% performance under strong noise (SNR ≤ 0.05). This approach outperforms existing methods in precision and resilience.

This method provides a standardized, interpretable, and scalable solution for quantitatively evaluating silicone rubber degradation in high-voltage power systems. Compared with other aging assessment approaches, the proposed method offers the advantages of simple operation and high accuracy, while enabling a more comprehensive analysis of the functional groups involved in the aging of silicone rubber. By automating the assessment process based on reproducible spectral measurements, it minimizes human error and contributes to the safe, efficient operation of electrical infrastructure.

However, the current model still relies on data augmentation, which presents certain limitations. In the future, this model will be integrated into the State Grid Corporation of China’s composite insulator inspection platform, where it will leverage the platform’s extensive sampling data to continuously improve the model and further enhance its accuracy and generalization ability. At the same time, improvements to existing handheld FTIR spectrometers will be pursued to enable reliable in situ detection of composite insulators. These developments will substantially advance operation and maintenance practices in the power industry and contribute to ensuring the safe and stable operation of power systems.

## Figures and Tables

**Table 1:** Silicone rubber aging class preliminary classification. The HC grade is determined using the spray grading method, with HC1 representing the best hydrophobicity and HC7 representing the worst hydrophobicity.

Aging Class	Composite Characteristics	Number of Samples
Light Aging (0)	Water-repellent grades HC1-HC3, high elasticity, glossy surface, hardness below 70 Shore A, no visible roughness or chalking (Overall performance close to that of new silicone rubber products)	225
Medium Aging (1)	Water-repellent grades HC4-HC7 with reduced gloss, hardness 70-80 Shore A, increased roughness or water-repellent grades HC4-HC5 with complete loss of gloss.	290
Severe Aging (2)	Water repellent grades HC6-HC7 with complete loss of surface gloss, hardness above 80 Shore A and high roughness or significant chalking of the silicone rubber surface.	225

**Table 2:** Wave number of absorption by a functional group.

Functional Group	Wave Number (${\mathrm{cm}}^{-1}$)
-OH	3200–3700
C-H_SV	2950–2975
C-H_BV	1200–1270
Si-O-Si	900–1168
Si-CH_3_	765–870

**Table 3:** The hyperparameters optimized by the base learners.

Models	Hyperparameters
KNN	’n_neighbors’: 1, ’weights’: ’distance’, ’metric’: ’minkowski’, ’leaf_size’: 47, ’algorithm’: ’brute’, ’p’: 4
SVM	’C’: 10.659, ’kernel’: ’rbf’, ’gamma’: 4.052
RF	’max_depth’: 10, ’max_features’: 1, ’min_samples_leaf’: 1, ’min_samples_split’: 4, ’n_estimators’: 80
GBDT	’n_estimators’: 83, ’learning_rate’: 0.096, ’max_depth’: 7, ’min_samples_split’: 15

**Table 4:** Performance on each class of the model.

Class	Precision	Recall	F1 Score	Accuracy
Light	0.96	0.92	0.96	0.9617
Medium	0.96	0.96	0.93	
Severe	0.96	0.94	0.94	

**Table 5:** SSE for each model.

Model	SSE
Without RF, SVM	0.2253
SVM	0.0538
Ensemble	0.00148

**Table 6:** Results of ablation experiments.

Removed Items	Accuracy (%)	Performance Changes (%)
Data Enhancement	83.10	−13.07
KNN	94.81	−1.36
SVM	94.01	−2.16
RF	93.29	−2.88
GBDT	92.64	−3.53
KNN, SVM	86.43	−9.74
KNN, RF	87.38	−8.79
KNN, GBDT	88.62	−7.55
SVM, RF	82.74	−13.43
SVM, GBDT	84.34	−11.83
RF, GBDT	87.94	−8.23
Weighting analysis	87.75	−8.42
Weighting analysis, loss function	85.88	−10.29

## Data Availability

The data presented in this study are available on request from the corresponding author. (Due to the involvement of commercial confidentiality with the SGCC, some data have not been made publicly available).
